# Efficacy of Dimethyl Trisulfide on the Suppression of Ring Rot Disease Caused by *Botryosphaeria dothidea* and Induction of Defense-Related Genes on Apple Fruits

**DOI:** 10.3389/fmicb.2022.796167

**Published:** 2022-02-07

**Authors:** Meng Sun, Yanxin Duan, Jun Ping Liu, Jing Fu, Yonghong Huang

**Affiliations:** ^1^College of Horticulture, Qingdao Agricultural University, Qingdao, China; ^2^Laboratory of Quality and Safety Risk Assessment for Fruit (Qingdao), Ministry of Agriculture and Rural Affairs, Qingdao, China; ^3^National Technology Centre for Whole Process Quality Control of FSEN Horticultural Products (Qingdao), Qingdao, China; ^4^Qingdao Key Laboratory of Modern Agriculture Quality and Safety Engineering, Qingdao, China

**Keywords:** *Allium tuberosum*, *Botryosphaeria dothidea*, biocontrol, defensive genes, fruit quality

## Abstract

Apple ring rot caused by *Botryosphaeria dothidea* is prevalent in main apple-producing areas in China, bringing substantial economic losses to the growers. In the present study, we demonstrated the inhibitory effect of dimethyl trisulfide (DT), one of the main activity components identified in Chinese leek (*Allium tuberosum*) volatile, on the apple ring rot on postharvest fruits. In *in vitro* experiment, 250 μL/L DT completely suppressed the mycelia growth of *B. dothidea*. In *in vivo* experiment, 15.63 μL/L DT showed 97% inhibition against the apple ring rot on postharvest fruit. In addition, the soluble sugar content, vitamin C content, and the soluble sugar/titratable acidity ratio of the DT-treated fruit were significantly higher than those of the control fruit. On this basis, we further explored the preliminary underlying mechanism. Microscopic observation revealed that DT seriously disrupted the normal morphology of *B. dothidea*. qRT-PCR determination showed the defense-related genes in DT-treated fruit were higher than those in the control fruit by 4.13–296.50 times, which showed that DT inhibited apple ring rot on postharvest fruit by suppressing the growth of *B. dothidea*, and inducing the defense-related genes in apple fruit. The findings of this study provided an efficient, safe, and environment-friendly alternative to control the apple ring rot on apple fruit.

## Introduction

Apple is one of the most critical and popular fruits around the world. However, apple ring rot caused by the latent pathogen *Botryosphaeria dothidea* has seriously threatened apple production in recent years. The pathogen infects branches and trunks, resulting in wart-like symptoms around lenticels. As the disease aggravates, the infected trunks and shoots die. *B. dothidea* also infects apple fruit, causing slightly sunken lesions with alternating tan and brown rings. Subsequently, the diseased fruit rots quickly with a sour smell and oozes brown mucus sometimes (Tang et al., 2012; Bai et al., 2015). The decayed fruit proportion caused by the disease usually ranges between 10 and 20% each year. However, it may reach 70% in seasons with conditions conducive to fungal development (Zhao et al., 2016).

In addition to apple fruits, *B. dothidea* also infects various fruit such as fig (*Ficus carica*) (Wang et al., 2020), olive (*Olea europaea* L.) (Korukmez et al., 2019), sweet cherry (*Prunus avium* L.) (Zhang et al., 2019), pomegranate (*Punica granatum* L.) (Gu et al., 2020), apricot (*Prunus armeniaca* L.) (Huang et al., 2019b), mulberry (*Morus alba* L.) (Huang et al., 2019a), pear (*Pyrus bretschneideri* Rehd.) (Sun et al., 2020), avocado (*Persea americana*) (Qiu et al., 2020), and kiwifruit (*Actinidia chinensis*) (Wang et al., 2021). Therefore, it is indispensable to develop efficient ways to prevent and control the spread of *B. dothidea*.

Cultivating resistant varieties is the most economical and effective approach for controlling apple ring rot caused by *B. dothidea*. Unfortunately, the major commercial cultivars like red fuji, golden delicious, gala, and red delicious are highly susceptible to *B. dothidea* (Guan et al., 2015). Therefore, fungicide application is still the primary method to control apple ring rot worldwide (Fan et al., 2016, 2019). Previous studies showed that synthetic fungicides including fludioxonil, fluazinam, pyrisoxazole (Song et al., 2018), difenoconazole, tebuconazole, prochloraz, trifloxystrobin (Dai et al., 2017), tebuconazole (Fan et al., 2016), and pyraclostrobin (Fan et al., 2019) exhibit great potential for inhibiting the apple ring rot. However, the overuse of synthetic chemical pesticides leads to the development of fungicide resistance and causes environmental pollution and health problems.

Therefore, it increasingly stimulates the research on a safer and more eco-friendly alternative means to control plant disease. Naturally derived bioactive compounds are one of the most promising ecological alternatives and have advantages over synthetic fungicides. Previous studies showed that pure monoterpenes in plants including cuminaldehyde, geraniol, and β-citronellol have promising antifungal effects against *B. dothidea* (Zhang et al., 2018). In addition, 32 essential oil monomers exhibit a varying inhibitory effect on *B. dothidea* (Li et al., 2021). The lemon (*Citrus limon* L.) essential oil contains limonene (61.68%), neral (21.66%), γ-pinene (10.23%), γ-terpinene (6.42%) and exhibits potent antifungal activity against *B. dothidea* (Ammad et al., 2018). 4-hydroxycinnamic acid from Moso bamboo (*Phyllostachys pubescens*) leaf shows good antifungal activity to *B. dothidea* (Liao et al., 2021). Matrine significantly inhibits the mycelial growth of *B. dothidea* (Pan et al., 2019). Chelidonine in *Chelidonium majus*, shows intense fungal activity against *B. dothidea* (Pan et al., 2017). Furocoumarins, phenylethyl esters, alcarindiol, and sesquiterpenoid from *Notopterygium incisum* exhibit antifungal activities against *B. dothidea* (Xiao et al., 2018). Several active components, including schisanhenol B, schizandrin A, schizarin D, schizandrin B, gomisin L, schizandrol A, schizandrol B, isoschizandrin, schisanlignone A, kadsulignan M identified from *Schisandra chinensis*, inhibit hyphal growth of *B. dothidea* and significantly inhibited the apple ring rot on fruits (Yi et al., 2016).

Our preliminary study found that the Chinese leek (*Allium tuberosum*) extract exhibited potent inhibition on the mycelia growth of *B. dothidea*, thereby significantly suppressing the incidence of apple ring rot on detached shoots and postharvest fruit (Zhao et al., 2017). However, the exact mechanism remains elusive. Therefore, in the present study, we tried to demonstrate the antifungal activity of dimethyl trisulfide (DT), one of the essential components in Chinese leek volatile, against apple ring rot on postharvest fruit. On this basis, we also attempted to explore the underlying mechanism involved in the DT inhibitory effect on apple ring rot from two aspects: fungus *B. dothidea* and the apple fruits.

## Materials and Methods

### Experimental Material, Fungus, and Reagents

The apple fruit (*Malus domestica* Borkh. cv. Red Fuji) used in the experiments was purchased in the local supermarkets. The fruit with uniform sizes, no disease spots, and no mechanical damages was selected for the experiments. DT was provided by Cheng Du Micxy Chemical Co., Ltd., Chengdu, China. The fungal *B. dothidea* was isolated from diseased fruit in a apple orchards in Yantai city and identified by Sangon Biotech (Shanghai, China) (Fu, 2021), and it was kept in potato dextrose agar (PDA) medium.

### Determination of the Inhibitory Effect of Dimethyl Trisulfide on the Mycelia Growth of *Botryosphaeria dothidea*

Various concentrations of DT (250, 125, 62.5, and 31.25 μL/mL) were prepared to verify the inhibitory effects of DT on the growth of *B. dothidea*. The experiment was performed referring to the previously published method with minor modifications (Kurt et al., 2011). Firstly, a mycelial disk (0.5 cm in diameter) was inoculated in the center of a Petri dish (9 cm in diameter, 70 mL in volume) containing 20 mL of PDA medium. After that, the various concentrations of DT (100 μL) were added onto the filter paper (2 cm × 2 cm) laid on the center of the Petri dish inner lid. Thus the actual DT concentrations in the Petri dish space were 500, 250, 125, and 62.5 μL/L, respectively. In addition, 100 μL sterilized water was used as control. The experiment was performed in six replicates. The inoculated Petri dishes were inverted and incubated at 28°C in the dark for 5 days. The fungal colony diameters were measured every day to evaluate the inhibitory effect of DT on mycelia growth. The area-under-curve (AUC) of fungal diameter was calculated using the following formula (Huang et al., 2012).


$$\text{AUC}=\sum_{i=1}^{n-1}\left[\frac{X_{i+1}+X_{i}}{2}\right]t_{i+1}-t_{i}\;\left(1\right)$$

*X* is the inhibition. *n* is the number of evaluations, and the (*t*_*i*-1_−*t*_*i*_) is the time interval (days) between two consecutive evaluations.

### The Inhibitory Effects of Dimethyl Trisulfide on the Incidence of Apple Ring Rot on Fruit

To assess the inhibitory effects of DT on the incidence of apple ring rot on fruit, we designed two experiments. The two experiments were the same except for the inoculation method.

#### Experiment 1

The healthy apple fruit was sterilized with 75% alcohol and then washed with sterilized distilled water three times. A mycelial disk (0.5 cm in diameter) was inoculated into a small hole (0.5 cm in diameter and 0.5 cm in depth) which was made at the fruit’s equator with a sterilized hole puncher. Two inoculated fruits (0.5 L in volume) were placed into plastic boxes (4.5 L in volume). Then, 2 mL of DT at various concentrations (125, 62.5, and 31.25 μL/mL) were added to the petri dish lid placed at one of the corners of the plastic box. The same volume of sterilized water was used as a control. Thus the final DT concentration in the plastic box space was 62.5, 31.25, and 15.63 μL/L, respectively. The plastic boxes were sealed and incubated at 28°C in the dark. The experiment was repeated four times. The disease symptom was recorded, and the disease spot diameter was measured every day to evaluate the inhibitory effect of DT against apple ring rot. The AUC of disease spot diameters was calculated as the formula (1).

#### Experiment 2

Five mycelial disks (0.5 cm in diameter) were inoculated in 50 mL potato dextrose (PD) broth medium and incubated in a shaker at 28°C, 200 r/min for 48 h. The obtained mycelium pellets were broken into homogenate by ultrasound. The healthy apple fruit was sterilized with 75% alcohol and washed with sterilized distilled water three times. Then the fruit was inoculated with *B. dothidea* by dipping into the fungal homogenate for 15 min. Two inoculated apple fruit (0.5 L in volume) were placed in the plastic boxes (4.5 L in volume). Then 2 mL of DT at various concentrations (125, 62.5, and 31.25 μL/mL) were added into the petri dish lid placed at one of the corners of the plastic box. The same volume of sterilized water was used as a control. Thus the final DT concentration in the plastic boxes was 62.5, 31.25, and 15.63 μL/L, respectively. The plastic boxes were sealed and incubated at 28°C in the dark. The disease symptom was observed every day to evaluate the inhibitory effect of DT on apple ring rot.

### Effects of Dimethyl Trisulfide on the Mycelial Morphology of the *Botryosphaeria dothidea*

A mycelial disk (0.5 cm in diameter) was inoculated on the center of the cellophane paper laid on the PDA medium (20 mL) contained in Petri dish (9 cm in diameter, 70 mL in volume). The Petri dishes were inverted and incubated at 28°C in the dark until the mycelium grew to half the medium surface. Then 100 μL DT (250 μL/L) was added onto the filter paper (2 cm × 2 cm) laid on the Petri dish’s inner lid center. Thus, the actual DT concentration in the Petri dish space was 500 μL/L. 100 μL sterilized distilled water was used as a control. All the Petri dishes continued to be incubated at 28°C in the dark for 24 h. Then the cellophane paper with mycelia was cut into 1-cm-wide pieces and spread onto the glass slide. The mycelial morphology was observed using an inverted microscope (EVOS Auto 2, Thermo Fisher Scientific, San Jose, CA, United States) at 40×.

### Effects of Dimethyl Trisulfide on the Internal Qualities of the Apple Fruit

The experiment consisted of four treatments, viz. (1) CK: The apple fruit was soaked in PD broth medium for 15 min. (2) Bd: The apple fruit was soaked in *B. dothidea* culture for 15 min. (3) DT: The apple fruit was soaked in 62.5 μL/L DT for 15 min. (4) DT + Bd: The apple fruit was soaked in 62.5 μL/L DT for 15 min, then were soaked in *B. dothidea* culture for 15 min following air-drying at room temperature for 1 h. All the fruits were put into plastic boxes and incubated at 28°C in the dark. Apple fruit was sampled at 0, 3, 6, 9, and 12 days to determine the dynamic changes of internal fruit quality indices, including total soluble solid (TSS), soluble sugar (SS), titratable acidity (TA), and vitamin C (VC), total soluble solid/titrable acidity (TSS/TA), and soluble sugar/titrable acidity (SS/TA). TSS content was determined using a PAL-1 type sugar concentration detector (ATAGO, Japan). SS content was determined using the anthrone colorimetric method. The TA content was determined by NaOH titration. VC content was determined by 2, 6-dichloroindophenol colorimetric (Yang L. et al., 2020). The experiment was performed in three replicates. The AUC of internal quality indices was calculated as the formula (1), where *X* is the contents of fruit internal quality indices.

### Determination of the Expression of Defense Related-Genes Responded to Dimethyl Trisulfide

As previously described, four treatments (CK, DT, Bd, and Bd + DT) were performed. All the fruits were put into plastic boxes and incubated at 28°C in the dark. The disease symptoms were observed every day. 12 days later, a slight disease symptom emerged on the Bd-treated apple fruit. All the treated and control apple fruit were peeled around the equator with a sterilized paring knife. All the samples were stored at −80°C for later use.

The expressions of the defense-related genes including phenylalanine ammonia-lyase (PAL), glucanase_1 (GLU-1), glucanase_2 (GLU-2), glucanase_3 (GLU-3), peroxidase_1 (POD-1), peroxidase_2 (POD-2), polyphenol oxidase (PPO), catalase (CAT), and endochitinase (CHI) were determined by quantitative real-time PCR (qRT-PCR). We drew the gene sequence information of the nine genes mentioned above from the apple genome (ASM211411v1). The special primers were designed using primer 5 (Table 1) and synthesized by Sangon Biotech (Shanghai) Co., Ltd., Shanghai, China.

**TABLE 1:** The primer sequences of defense-related genes and internal reference genes.

GeneName	Gene ID	Primers	Sequence (5′–3′)
PPO	NM_001319261.1	PPO -F	TCATGGCTCTTCTTCCCGTT
		PPO -R	GATTGGCAGATCGGAGCTTG
CHI	NM_001293894.1	CHI-F	GAGACTACTGGAGGATGGGC
		CHI-R	ATCCTTTCCGATTGCTTGGC
CAT	XM_008375181.3	CAT-F	CCTCGTGGTTTTGCAGTGAA
		CAT-R	GGAAGGTGAACATGTGCAGG
POD-1	XM_029099288.1	POD1-F	TCCTGTGCTGACATTTTGGC
		POD1-R	AATCTACATTTTGCCCGGCC
POD-2	XM_029091729.1	POD2-F	GTTGCACTCGTGATGTTGGT
		POD2-R	CAATCATGGAAGTGGAGGCG
PAL	XM_008368428.3	PAL -F	GCAGAGCAACACAACCAAGA
		PAL -R	CGTTAAAGCCCATGGTGAGG
GLU-1	NM_001293850.1	GLU1-F	GAGCCAGTGATTCAACGGAC
		GLU1-R	TGGAGGTTACTTCCAGGCAG
GLU-2	XM_008343526.2	GLU2-F	ATGTGGTGGCATGTGAGAGA
		GLU2-R	CTAGCAAACCCGACATCAGC
GLU-3	XM_029095631.1	GLU3-F	CCCTGATTCCAACCTTGCTG
		GLU3-R	AAATCCCTTGTCCGGTCCAT
Actin	XM_008393049.3	Actin-F	CTTCAATGTGCCTGCCATGTAT
		Actin-R	AATTTCCCGTTCAGCAGTAGTG

*PPO, polyphenol oxidase; CHI, endochitinase; CAT, catalase; POD-1, peroxidase_1; POD-2, peroxidase_2; PAL, phenylalanine ammonia-lyase; GLU-1, glucanase_1; GLU-2, glucanase_2; GLU-3, glucanase_3.*

According to RNAprep Pure Plant Plus Kit [Tiangen Biotech (Beijing) Co., Ltd., Beijing, China], the RNA was extracted from the fruit samples. And the cDNA was synthesized using HiScript^®^ III RT SuperMix for qPCR (+gDNA wiper) (Nanjing Vazyme Biotech Co., Ltd., Nanjing, China). qRT-PCR was performed using ABI7500 Thermal Cycler (Applied Biosystems, Foster City, CA, United States) to detect the expressions of the nine defense-related genes aforementioned. The reaction system (10 μL) contained 5 μL of 2× ChamQ SYBR Color qPCR Master Mix (Nanjing Vazyme Biotech Co., Ltd., Nanjing, China), 0.2 μL of each primer, 0.2 μL of the 50× ROX Reference Dye I, 1 μL of cDNA, 3.4 μL of the ddH_2_O. The reaction conditions were as follows: 95°C for 30 s, followed by 40 cycles of 95°C for 10 s and 60°C for 30 s, then 95°C for 15 s, 60°C for 1 min and finally 95°C for 15 s. Actin was used as the internal reference gene, and the relative expression was calculated by 2^–ΔΔ*CT*^ method (Livak and Schmittgen, 2001). The experiment was performed in three replicates.

### Statistical Analysis

Experimental data were analyzed using standard analysis of variance (ANOVA) followed by least significant difference tests (*p* < 0.05) using the software statistical analytical system (SAS 9.0). Standard errors were calculated for all mean values.

## Results

### Dimethyl Trisulfide Inhibited the Mycelial Growth of *Botryosphaeria dothidea*

**FIGURE 1:**
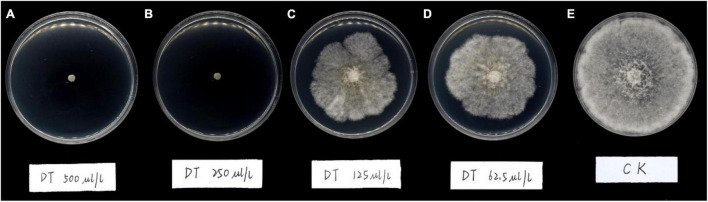
The mycelial growth of *Botryosphaeria dothidea* on PDA medium containing various concentrations of dimethyl trisulfide for 5 days. **(A)** 500 μL/L, **(B)** 250 μL/L, **(C)**: 125 μL/L, **(D)** 62.5 μL/L, **(E)** control.

**FIGURE 2:**
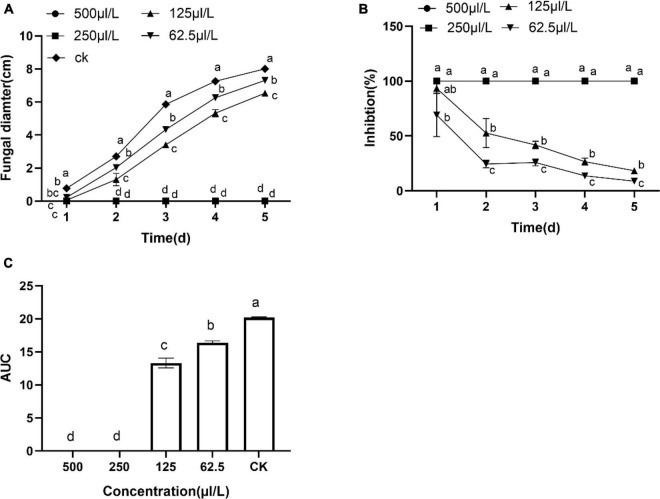
The dimethyl trisulfide inhibition on the mycelia growth of *Botryosphaeria dothidea*. **(A)** The colony diameters of *Botryosphaeria dothidea* treated by the various concentration of dimethyl trisulfide. **(B)** The inhibition of various concentrations of dimethyl trisulfide against mycelia growth of *Botryosphaeria dothidea*. **(C)** The area-under-curve (AUC) of fungal colony diameters treated by various concentrations of dimethyl trisulfide. Different lowercase letters indicate a significant difference between treatments (*P* < 0.05).

The results showed DT strongly inhibited the mycelia growth of the *B. dothidea*. The mycelia growth of the control was initiated on the first day and rapidly grew on successive days. On the fifth day, the fungal mycelia completely covered the whole surface of the Petri dish. But the DT-treated mycelia remained unchanged or grew slower than the control (Figure 1). Statistics showed that the colony diameters reduced with the increase of the DT concentration (Figure 2A). The inhibitory effect increased with DT concentration but decreased with incubation time. During the whole experimental period, 500 and 250 μL/L DT exhibited 100% inhibition against *B. dothidea*. 125 and 62.5 μL/L DT showed 93.5 and 69.0% inhibition on the first day, which sharply decreased to 18.3 and 8.75% on the fifth day, respectively (Figure 2B). The AUC of fungal colony diameters treated by various DT concentrations were reduced by 18.9–100% compared to that of control (*P* < 0.0001) (Figure 2C).

### Dimethyl Trisulfide Disrupted the Mycelial Morphology of *Botryosphaeria dothidea*

**FIGURE 3:**
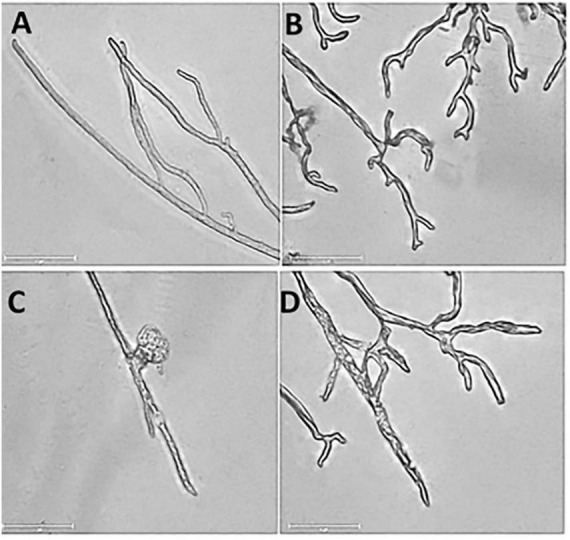
The mycelial morphology of *Botryosphaeria dothidea*. **(A)** The mycelia of the untreated control. **(B–D)** The mycelia treated with 500 μL/L dimethyl trisulfide for 24 h.

Under a microscope, the control mycelia had complete morphology with uniform thickness, smooth surface, slender and full shape (Figure 3A). However, DT seriously deformed the normal mycelial morphology. The outline of the DT-treated mycelia became blurred, with more bifurcation at the top, similar to the chicken feet. And the mycelia also became curved and wrinkled. The front end expanded, forming a tumor-like structure. Some mycelia ruptured and dissolved, and the contents spilled (Figures 3B–D).

### Dimethyl Trisulfide Inhibited the Incidence of Apple Ring Rot Disease Symptoms on Fruit

#### Experiment 1

**FIGURE 4:**
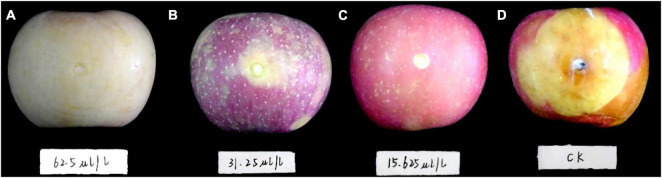
The apple fruit was first inoculated with *Botryosphaeria dothidea* disk, subsequently treated with dimethyl trisulfide, then cultured at 28°C in the dark for 5 days. Apple fruits with different treatments exhibited varying degrees of disease symptoms. **(A)** 62.5 μL/L, **(B)** 31.25 μL/L, **(C)** 15.63 μL/L, **(D)** control.

**FIGURE 5:**
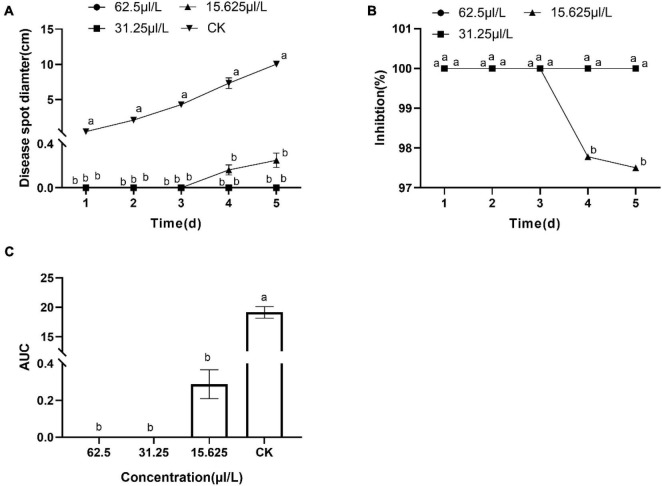
The inhibitory effect of dimethyl trisulfide on the apple ring rot on apple fruit. **(A)** The disease spot diameter on apple fruits treated by various concentrations of dimethyl trisulfide. **(B)** The inhibition of various concentrations of dimethyl trisulfide against the apple ring rot on fruit. **(C)** The area-under-curve (AUC) of disease spot diameter on fruit treated by the various concentration of dimethyl trisulfide. Different lowercase letters indicate a significant difference between different treatments (*P* < 0.05).

The first 2 days after inoculation, disease spots gradually appeared around the control fruit’s inoculating points. Three days later, the disease spots began to grow and developed rapidly. On the fifth day, the fruit was utterly rotten with a thick exudation on the surface and a sour smell. However, DT-treated apple fruit showed no disease symptoms during the early days of the experiment. After 5 days of inoculation, only mild disease spots appeared on the apple fruit treated with the low concentration DT (15.63 μL/L) (Figure 4). Statistical analysis showed that the disease spot diameter of the control fruit reached up to 10.03 cm on the fifth day, while that of fruit treated with a low DT concentration (15.63 μL/L) was 0.25 cm (Figure 5A), indicating a 97.5% inhibition. High DT concentrations (62.5 and 31.25 μL/L) exhibited 100% inhibition against the apple ring rot (Figure 5B). The AUC of disease spot diameter on fruit treated by the various DT concentrations was reduced by 98.5–100% compared to that of control (*P* < 0.0001) (Figure 5C).

#### Experiments 2

**FIGURE 6:**
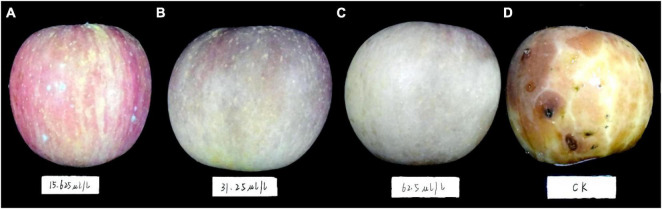
The apple fruit was first dipped in a *Botryosphaeria dothidea* culture for 15 min, subsequently exposed to various concentrations of dimethyl trisulfide, then cultured at 28°C in the dark for 4 weeks. Apple fruits with different treatments exhibited varying degrees of disease symptoms. **(A)** 15.63 μL/L, **(B)** 31.25 μL/L, **(C)** 62.5 μL/L, **(D)** control.

Only 1 day after inoculation, sparse mycelial colonies with different sizes appeared on the control fruit. During the first week, the colonies successively emerged and enlarged quickly, finally covering the whole fruit surface. The disease spots gradually appeared across the fruit surface during the second week. They progressively expanded and joined together later, eventually leading to fruit decay during the third and fourth weeks. However, apple fruits treated with a low DT concentration (15.63 μL/L) showed no symptoms during the first week. During the second week, an average of 3 sparse mycelial colonies appeared on the fruit. On average, 10 sparse mycelial colonies appeared on the fruit during the third and fourth weeks. However, the apple fruit treated with higher DT concentrations (>31.25 μL/L) showed no obvious disease symptom and exhibited 100% inhibition against the apple ring rot throughout the experiment period (Figure 6).

### Dimethyl Trisulfide Enhanced the Apple Fruit Quality

**FIGURE 7:**
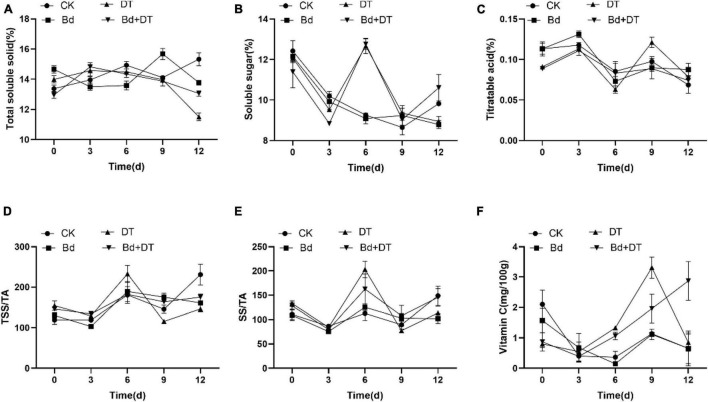
The dynamic changes of internal quality indexes including total soluble solid **(A)**, soluble sugar **(B)**, titratable acidity **(C)**, TSS/TA **(D)**, SS/TA **(E)**, and vitamin C **(F)** in the different treatment fruit.

**FIGURE 8:**
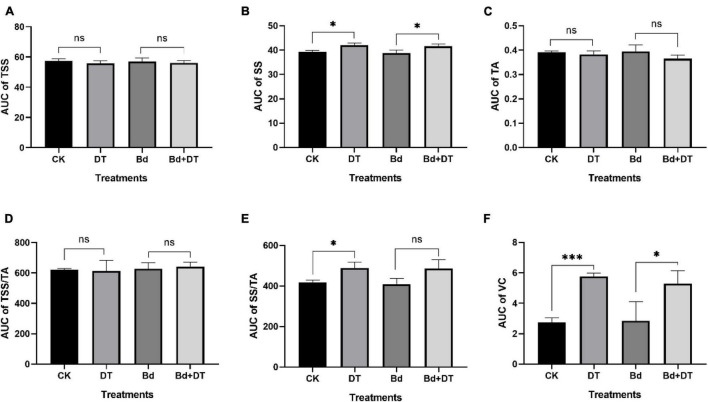
The area-under-curve (AUC) of internal quality indexes including total soluble solid **(A)**, soluble sugar **(B)**, titratable acidity **(C)**, TSS/TA **(D)**, SS/TA **(E)**, and vitamin C **(F)** the different treatment fruit. *P < 0.05, ^***^P < 0.001, ns *P* > 0.05.

All the six fruit quality indices of the four treatments (CK, DT, Bd, and DT + Bd) showed an up-and-down trend, fluctuating with prolonged time (Figure 7). The AUC of fruit quality indices was used to evaluate DT’s overall effect on the fruit quality. Compared to the control fruit (CK), DT treatment (DT) increased the SS content, SS/TA ratio and VC content by 4.22% (*P* = 0.0124), 16.59% (*P* = 0.0209), and 109.80% (*P* < 0.0001), respectively. Compared to the fruit inoculated with Bd (Bd), DT treatment (DT + Bd) significantly increased VC content by 86.13% (*P* = 0.0499), SS content by 3.67% (*P* = 0.0346), and also enhanced SS/TA ratio by 19.05% (*P* = 0.0581) (Figure 8).

### Dimethyl Trisulfide Induced the Expression of Defense-Related Genes

**FIGURE 9:**
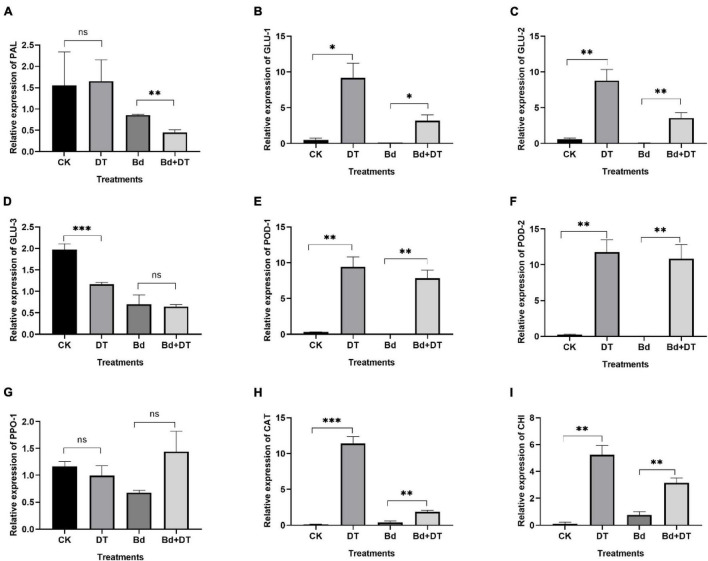
The expression analysis of various defense-related genes including phenylalanine ammonia-lyase **(A)**, glucanase_1 **(B)**, glucanase_2 **(C)**, glucanase_3 **(D)**, peroxidase_1 **(E)**, peroxidase_2 **(F)**, polyphenol oxidase **(G)**, catalase **(H)**, endochitinase **(I)** in the DT-treated fruit. *P < 0.05, ^**^P < 0.01, ^***^P < 0.001, ns *P* > 0.05.

Dimethyl trisulfide induced all the detected genes in apple fruit, six (GLU-1, GLU-2, POD-1, POD-2, CHI, and CAT) of which were significantly up-regulated (Figure 9). Compared with the control (CK), the six genes in DT-treated apple fruit (DT) were up-regulated by 14.91 (GLU-2) to 84.09 (CAT) times, with an average of 39.09 times. Compared to fruit inoculated with *B. dothidea* (Bd), the six genes in DT-treated apple fruit (Bd + DT) were increased by 4.13 (CHI) to 296.50 (POD-2) times, averaged 95.24 times. These results revealed that the DT markedly induces apple fruit’s defense-related genes whether or not they were inoculated with *B. dothidea.*

## Discussion

Nowadays, extensive application of various synthetic fungicides is still the primary management strategy that effectively controls apple ring rot. But the pesticide residue resulted in potential harm to the environment, animals, even humans. Therefore, due to natural products’ environment-friendly and low toxicity, developing natural fungicides from plants has attracted more attention worldwide. In the present study, we revealed that DT, one of the main components from Chinese leek, significantly suppressed apple ring rot on postharvest fruit. DT naturally existed in Chinese leek and other *Allium* plants. Plants of the *Allium* family, such as garlic (*Allium sativum*), onion (*Allium cepa*), and Chinese leek, have been cultivated for food since earliest times (Munday et al., 2003). And DT is widely distributed in foods and beverages such as broccoli, milk, cheese, whiskey, hineka, beer, and wine (Gijs et al., 2002; Isogai et al., 2009). Therefore, using DT to control apple ring rot is efficient and safe for humans, animals, and the environment, providing a possible way to control apple ring rot.

In the present study, we found that the DT inhibition at low concentration on the mycelia growth sharply decreased with the prolonged experimental time. For example, 125 and 62.5 μL/L DT showed 93.5 and 69.0% inhibition on the first day, but they decreased to 18.3 and 8.75% on the fifth day, respectively. That is because DT was highly volatile and quickly escaped from the Petri dishes into the air in a short period. In addition, DT interacted with the fungal mycelia and partly decomposed. The escape or decomposition rapidly decreased DT concentration in the petri dish. However, the remaining concentration was not sufficient to inhibit mycelial growth. To solve this problem in practical application, DT can be prepared into the slow-release formulation to maintain sustainable high inhibition.

To prove the inhibitory effect of DT on apple ring rot on postharvest fruit, we designed two inoculation methods, inoculating the apple fruit with a mycelial disk and socking the apple fruit in the *B. dothidea* culture for 15 min. The two experiments revealed that DT significantly suppressed the incidence of the disease. The high concentration (62.5 and 31.25 μL/L) of DT completely inhibited the disease, and the low concentration (15.63 μl/L) also showed more than 90% inhibition. However, the red skin color of apple fruit treated with high concentration (62.5 and 31.25 μL/L) DT somewhat faded, but that of the apple fruits treated with low concentration (15.63 μl/L) DT was perfectly maintained. Therefore, we suggest using lower DT concentration to treat apple fruit in practical application, dramatically maintaining the fruit color and preventing disease. Furthermore, the pathogen amount carried on the field fruit in the practical production was far lower than those artificially inoculated in our experiment. Thus, our present study demonstrated the potential application of DT as an alternative strategy against apple ring rot caused by *B. dothidea*.

The present study explored the underlying mechanism from the fungal *B. dothidea* aspect and the apple fruit aspect. From the findings in the present study, we deduced that DT inhibits the disease incidence by suppressing the growth of *B. dothidea* and inducing defense-related genes in apple fruit.

On the one hand, DT caused severe disruption to *B. dothidea* mycelia, leading to its slow growth or even death, further decreasing its vigor and amounts, thereby reducing the infection probability and ultimately suppressing the incidence of apple ring rot on apple fruit. The previous study showed that the essential oil aromatic plants, including origanum (*Origanum syriacum* L.), lavender (*Lavandula stoechas* L.), and rosemary (*Rosmarinus officinalis* L.), significantly inhibit the growth of *Phytophthora infestans* (Soylu et al., 2006) and *Botrytis cinerea* (Soylu et al., 2010) by causing considerable morphological degenerations of the fungal mycelia. Origanum contains carvacrol (79.8%) and *p*-cymene (8.2%), lavender contains camphor (20.2%), 1,8-cineole (35.5%), α-thujone (15.9%) and fenchone (13.5%), rosemary contains borneol (20.4%), camphor (19.5%), 1,8-cineole (17.4%) and linalool (6.1%) as the main components (Soylu et al., 2006). These components interfere with the enzymatic reactions of wall synthesis, resulting in considerable morphological alterations in mycelia such as cytoplasmic coagulation, vacuolations, hyphal shriveling, and protoplast leakage, finally affecting the fungal morphogenesis and growth (Rasooli et al., 2006; Soylu et al., 2006, 2010). Therefore, we believe the direct effect of DT on fungal *B. dothidea* mycelium may be an important factor inhibiting the ring rot disease on apple fruit.

On the other hand, DT significantly induced defense-related genes, including GLU-1, GLU-2, POD-1, POD-2, CHI, and CAT, by 4.13 to 296.49 times compared to the control. Previous studies revealed that the defense-related response played an essential role in improving the disease resistance of plants. GLU enhances the tolerant of rough lemon rootstock against foot rot (Sandhu et al., 2019), improves the inhibition of rice against *Rhizoctonia solani* (Kumar et al., 2018), reduces the rice sheath blight (Kaur et al., 2021), and confers tea tree resistance against blister blight disease (Singh et al., 2018). POD is involved in the resistance response of coffee varieties to *Colletotrichum kahawae* (Diniz et al., 2019), potato to *P. infestans* (Yang Y. et al., 2020), *Arabidopsis thaliana* to *Botrytis cinerea*, *Colletotrichum higginsianum*, and *Pectobacterium carotovorum* (Zhao et al., 2019), *Citrus sinensis* to citrus bacterial canker (Li et al., 2020). CAT contributes maize to resistance against maize chlorotic mottle virus infection (Jiao et al., 2021), *Nicotiana tabacum* against *Chilli veinal mottle virus* infection (Yang T. et al., 2020). CHI enhances tobacco tolerance against *B. cinerea* (Navarro-Gonzalez et al., 2019), *Arabidopsis thaliana* resistance against the bacterium *Xanthomonas campestris* pv. *campestris* (Xcc) (Santos et al., 2019), and tomato resistance against *B. cinerea* (Zheng et al., 2018). According to the above studies, the significantly up-regulated POD, GLU, CHI, and CAT were probably critical in enhancing apple fruit resistance against *B. dothidea* infection.

In practical application, we can spray DT on the tree or the fruit during the growing season to control apple ring rot on branches or fruit. After harvesting, we can spray DT on fruit, dip the fruits in DT solution, or place DT in the corner of the fruit box to prevent apple ring rot on postharvest fruit. Chinese leek contains a large amount of DT or other organic sulfides. The essential oil of Chinese leek leaves contains disulfide compounds (64.9%) and trisulphide compounds (18.9%), the flower contains trisulphide compounds (34.0%) and disulfide compounds (20.2%), the essential oil of rhizome contains trisulphide compounds (47.3%), and the seed contains disulfide (32.4%) and tetrasulfide (5.2%) as the main constituents (Hu et al., 2013). These sulfides contained in Chinese leek, including allyl methyl sulfide, methyl disulfide, dimethyl trisulfide, and methyl allyl trisulfide (Zhao et al., 2017), diallyl disulfide, diallyl trisulfide, and diallyl tetrasulfide (Rattanachaikunsopon and Phumkhachorn, 2009), also exhibit vigorous and widely antifungal or antibacterial activity. Our previous study showed that Chinese leek significantly reduced the banana *Fusarium* wilt (Huang et al., 2012) by intercropping or rotating crops. Therefore, we can also intercrop Chinese leek in the orchard to control apple ring rot in practice production.

Besides Chinese leek, other *Allium* plants, including *A. sativum* (Natasya-Ain et al., 2018), *A. cepa* (Balamanikandan et al., 2015; Ortega-Ramirez et al., 2017), *Allium stipitatum* (Karunanidhi et al., 2017), *Allium saralicum* (Zangeneh M.M. et al., 2019), *Allium noeanum* (Shahriari et al., 2019), *Allium saralicum* (Zangeneh A. et al., 2019), *Allium hirtifolium* (Ismail et al., 2013), are reported to possess extensive antifungal or antibacterial activity. They also contain a large number of organic sulfides with intense antifungal activity. For example, propyl-propane thiosulfinate, propyl-propane thiosulfonate (Sorlozano-Puerto et al., 2020), methyl allyl thiosulphinate, E-ajoene and Z-ajoene from garlic (*A. sativum*), elephant garlic (*Allium ampeloprasum*), and onion (*A. cepa*) (Hughes and Lawson, 1991), Allicin (Bhattacharya et al., 2019), diallyl disulfide (Jin et al., 2021) from garlic (*A. sativum*), also exhibit vigorous and widely antifungal or antibacterial activity. These *Allium* plants are an excellent natural resource to control plant disease. However, they have been ignored for many years. For example, after harvesting garlic bulbs, the plant is often discarded in the field bank and the ditch, resulting in waste and pollution. We can take good advantage of these valuable resources by burying them in the soil around the tree, which controls the disease incidence and reduces pollution.

Perfect measures to control postharvest fruit diseases must have two fundamental characteristics: to effectively control disease occurrence and maintain fruit quality. In the present study, we demonstrate that DT showed a strong inhibitory effect on apple ring rot and significantly enhances SS and VC contents and the SS/TA ratio of the apple fruit, which suggests that DT is an ideal alternative strategy against postharvest apple ring rot.

## Conclusion

In the present study, we demonstrated that DT, the main component of Chinese leek, significantly suppresses the mycelia growth of *B. dothidea* and inhibits the incidence of apple ring rot on postharvest fruit. Most importantly, DT significantly enhanced the soluble sugar content, vitamin C content, and soluble/titratable acidity ratio. We further found DT inhibited the apple ring rot by directly suppressing the pathogen growth and inducing the fruit’s defense-related response. Therefore, it provides an efficient, safer, and environment-friendly alternative to control the apple ring rot on apple fruit.

## Data Availability Statement

The original contributions presented in the study are included in the article/supplementary material, further inquiries can be directed to the corresponding author.

## Author Contributions

YH contributed to the conception of the study and revised the manuscript. MS and YD wrote the manuscript, performed the experiments, and collected the data. JF experimented and collected the data. JL performed qRT-PCR validation. All authors contributed to the article and approved the submitted version.

## Conflict of Interest

The authors declare that the research was conducted in the absence of any commercial or financial relationships that could be construed as a potential conflict of interest.

## Publisher’s Note

All claims expressed in this article are solely those of the authors and do not necessarily represent those of their affiliated organizations, or those of the publisher, the editors and the reviewers. Any product that may be evaluated in this article, or claim that may be made by its manufacturer, is not guaranteed or endorsed by the publisher.
